# Improvement of the Brazilian nutritional scenario despite the persistence of social inequalities

**DOI:** 10.1590/0102-311XEN136323

**Published:** 2023-10-23

**Authors:** Chessa K. Lutter

**Affiliations:** 1 RTI International, Research Triangle Park, U.S.A.

From 2006 to 2019, Brazil’s economy grew rapidly with its per capita income increasing nearly 51%, from USD 5,866 to USD 8,845 ^1^. At the same time, its birthrate declined nearly 6%, from 1.84 to 1.73 births per woman, respectively ^1^. Years of formal education among Brazilian women increased, sanitation systems improved, as did access to the internet. During this period, conditional cash transfers to low-income households also took millions of Brazilians out of poverty. Although the World Bank classifies Brazil as an upper-middle-income country, its Gini coefficient, a measure of income inequality, was 53.5 in 2019, indicating a high degree of income inequality ^2^. It is against these dramatic economic and demographic changes that the study by de Castro et al. ^3^, which documented changes in child nutrition from 2006 to 2019 and infant and young child feeding practices in 2019, are examined.

A striking finding is that despite the geographic, maternal schooling, and racial disparities that persist in Brazil, inequalities for stunting, vitamin A deficiency, exclusive breastfeeding, and continued breastfeeding in 2006 markedly decreased by 2019. Though not eliminated, the fact that the disparities in these important indicators diminished speaks to the likely importance of economic growth, educational opportunities in women, improved water and sanitation infrastructure, and a strong social safety net to greater child nutrition. Indeed, an earlier study by Monteiro et al. ^4^ found that the 50% reduction in stunting from 1996 to 2006/2007 was related to four factors; improvements in maternal education (25.7%) increased family purchasing power (21.7%), expansion of health care (11.6%), and improvements in sanitation (4.3%) ^4^. A second study by the same authors showed a significant reduction in the gap of stunting between children in the highest and lowest socioeconomic quintiles from 1974/1975 to 2006/2007, during which time the overall prevalence of stunting declined from 37.1% to 7.1% ^5^. Although it is dispiriting that nationally no further improvements in stunting from 2006/2007 to 2019 were observed, the fact that during this period it declined by half, from about 15% to 8% in the Brazilian Northeast (the poorest region in the country) is encouraging.

The notable improvement in exclusive and continued breastfeeding speaks to the efforts Brazil has made to normalize breastfeeding by comprehensive policies and programs, including strong legislation and monitoring of the International Code of Marketing of Breastmilk Substitutes, widespread implementation of the Baby Friendly Hospital Initiative, comprehensive maternal and paternal maternity leave, and strong community support programs ^6^. The data, however, also show that indicators do not always move in the same direction. For example, while continued breastfeeding increased among women in the highest education category, the prevalence of exclusive breastfeeding decreased. Interestingly, both surveys showed that exclusive breastfeeding was highest among this education group, whereas continued breastfeeding was highest among women in the lowest education group. This may stem from highly educated womens’ great awareness of the benefits of exclusive breastfeeding and their lifestyles being more conducive to this practice, such as formal sector jobs that protects these women by generous maternity leave policies, access to skilled lactation counseling should breastfeeding problems arise, and better support for household chores.

Although just under 40% of children are not receiving their minimum dietary diversity - defined as consuming five out of eight core food groups the day prior to the survey -, this is still far lower than the global average of 71% ^7^. Intake of meat or eggs is also higher; among young children in Brazil, 83% versus 45% globally. However, it is troubling that, in a country of such rich biodiversity and availability of fruits and vegetables, a quarter of children did not consume such foods.

The 50% reduction in the prevalence of anemia cannot be strictly compared due to methodological differences in assessment between the two surveys. At the same time, Brazil should be recognized for using venous blood to assess anemia in 2019, rather than capillary blood obtained by a skin prick as in 2006/2007. Venous blood is more challenging for large survey collections as parents are often reluctant to have blood drawn, but provides a far more accurate estimate of the prevalence of anemia ^8^.

The fact that nearly 90% of Brazilian children consumed an ultra-processed food the day preceding the survey is astonishing and shows the dramatically changing landscape of children’s dietary intake. Brazil is not alone in this matter. A recent analysis by United Nations Children’s Fund (UNICEF) based on nationally representative data from 47 low- and middle-income countries covering 51% of the global population of children aged below two years showed that such foods are frequently consumed globally every day in these countries. For example, about one-third of children 6-23 months of age in Ghana and Zimbabwe consumed a sugar-sweetened beverage the day prior to a survey ^7^. Nearly 30% of children in Nigeria consumed instant noodles and about 30% of children in Mexico and Nigeria had consumed biscuits/cakes.

Lastly, the study by Castro et al. ^3^ highlights a conundrum that those working in child nutrition must confront and address: that policies and programs to reduce child undernutrition are unable to inadvertently increase the risk of child obesity. Despite important reductions in anemia and vitamin A deficiency and improvements in exclusive breastfeeding and continued breastfeeding observed from 2006/2007 to 2019, rates of child obesity increased. Obesity during childhood increases the risk of obesity in adulthood and adults with obesity are at higher risk of diabetes, coronary heart disease, and a wide range of cancers, and mortality ^9^. Therefore, childhood represents a critical period to prevent noncommunicable diseases later in life.

The increase in the prevalence of overweight in children not only in high-income countries but also in low- and middle-income countries calls for nutrition policies that promote healthy growth and protect children from overconsuming foods with poor nutrition quality ^10^. While economic growth to enable families to purchase foods, access health care, and have better environmental sanitation is fundamental to improving child nutrition, different measures are needed to prevent obesity. These includes fiscal policies that can reduce the purchase of unhealthy foods ^11^ ^,^ ^12^, restrictions of the marketing of unhealthy foods and beverages to children ^13^, and easy to understand front-of-pack labeling ^14^ ^,^ ^15^. Although changes in the food environment are essential (broader cultural shifts such as what happened with breastfeeding), are also necessary to reduce the consumption of ultra-processed foods. Caregivers of children (usually their mothers) are increasingly employed in both formal and informal sectors and have multiple tasks in addition to cooking and feeding their families. The importance of convenience to make women’s lives easier must be recognized and policies and programs designed to inform not only on what not to feed their children but also on what to feed them must include foods that are easy to prepare, take on errands or to daycare, and to feed infants and toddlers.

The challenge to prevent childhood stunting and overweight are enormous. Given its long trajectory of policies and programs to reduce poverty, increase women’s education, and promote breastfeeding and child nutrition generally, Brazil can build on these experiences to tackle the problem of child obesity by innovative approaches and the continued measurement of progress and the remaining challenges.
